# Vitamin intake and periodontal disease: a meta-analysis of observational studies

**DOI:** 10.1186/s12903-024-03850-5

**Published:** 2024-01-20

**Authors:** Nannan Mi, Miaomiao Zhang, Zheng Ying, Xiaoping Lin, Ying Jin

**Affiliations:** Department of Stomatology, Shengjing Hospital of China Medical University, Shenyang, 110004 China

**Keywords:** Vitamin, Folate, Periodontal disease, Meta-analysis

## Abstract

**Objective:**

A meta-analysis was performed to assess the epidemiological correlation between dietary intake of various types of vitamin intake and the risk of periodontal disease.

**Methods:**

A comprehensive computerized search was conducted in eight databases, namely PubMed, Web of Science, Embase, Cochrane Library, China Biology Medicine Disc, CNKI, VIP, and WanFang Database, and a random effect model was applied to combine pooled odds ratio (ORs) with corresponding 95% confidence intervals (CIs) of the included studies, and the sensitivity analysis was performed to explore the impact of a single study on the comprehensive results.

**Results:**

We finally included 45 effect groups from 23 observational studies, with a total number of study participants of 74,488. The results showed that higher levels of vitamin A (OR: 0.788, 95% CI: 0.640–0.971), vitamin B complex (OR: 0.884, 95% CI: 0.824–0.948), vitamin C (OR: 0.875, 95% CI: 0.775–0.988), vitamin D (OR: 0.964, 95% CI: 0.948–0.981), and vitamin E (OR: 0.868, 95% CI: 0.776–0.971) intake all were negatively correlated with periodontal disease. After removing each study, leave-one-out sensitivity analysis indicated no significant change in the overall results of any of the five meta-analyses.

**Conclusions:**

The results from this meta-analysis demonstrated a negative association between high-dose vitamin A, vitamin B complex, vitamin C, vitamin D, and vitamin E consumption and the likelihood of developing periodontal disease, revealing the significant role of vitamins in preventing periodontal disease.

**Supplementary Information:**

The online version contains supplementary material available at 10.1186/s12903-024-03850-5.

## Background

Periodontal disease, a prevalent chronic condition, affects a significant number of individuals worldwide. The number of global epidemic cases of severe periodontal disease alone reached 796 million in 2017 [1]. In the United States, 42% of adults over the age of 30 had periodontal disease [2]. The prevalence of periodontal disease varied from 10.2% to 55.7% among Korean adults [3]. Similarly, among Chinese adults, the detection rate of clinical attachment loss ≥ 4 mm ranged from 33.2% to 74.2% across different age groups [4]. Periodontal disease not only leads to the destruction of periodontal tissue but also adversely affects human overall health and quality of life. Numerous studies have increasingly established a strong correlation between periodontal disease and various systemic illnesses, such as Alzheimer's disease, diabetes, rheumatoid arthritis, inflammatory bowel disease, premature birth, and preeclampsia [5, 6]. These findings emphasized the importance of maintaining good oral health and addressing periodontal disease to safeguard against potential complications in other areas of health.

Periodontal disease is an inflammatory disease dominated by bacterial infection. In addition, some potential risk factors such as genetic susceptibility, hormonal changes, smoking, and alcohol consumption also promote its progression. In recent years, many studies have explored the relationship between various types of vitamin intake and the risk of periodontal disease, but no consistent conclusions have been reached. Some studies have found that higher levels of vitamin A, vitamin B6, vitamin B12, folate, vitamin C, vitamin D, and vitamin E intake were negatively correlated with the incidence of periodontal disease [7, 8]. However, some studies suggested that there was no significant correlation between folate, vitamin C intake, and periodontal disease [9, 10]. Therefore, this study aimed to conduct a quantitative synthesis of existing observational studies on the relationship between vitamins and periodontal disease. Furthermore, it sought to explore the dose–response relationship between vitamin intake and the risk of periodontal disease. By doing so, this research intended to provide a more objective assessment of the correlation between vitamins and periodontal disease, thus offering scientific evidence for the exploration of the etiology of periodontal disease and the formulation of primary prevention measures.

## Materials and methods

Reporting of the present study consulted the Preferred Reporting Items for Systematic Reviews and Meta-Analyses (PRISMA) guideline [11]. The protocol of this meta-analysis was registered with the internationally registered system of systematic review and meta-analysis protocols (PROSPERO) (ID CRD42023440945).

### Literature search strategy

To comprehensively obtain relevant literature on vitamin consumption and the risk of periodontal disease, we searched 8 databases including PubMed, Web of Science, Embase, Cochrane Library, China Biology Medicine Disc, CNKI, VIP, and WanFang Database (up to December 31, 2022), the literature search formula in PubMed is: ((“Vitamins”[Mesh]) OR (((vitamin[Title/Abstract]) OR (folate[Title/Abstract])) OR (folic acid[Title/Abstract]))) AND ((“Periodontitis”[Mesh]) OR (((periodontal disease[Title/Abstract]) OR (periodontitis[Title/Abstract])) OR (periodontal infection[Title/Abstract]))).

### Inclusion criteria

The included articles in this study should meet the following criteria: (1) original article in the form of observational study that assessed the epidemiological association between vitamins and the risk of periodontal disease, (2) the study participants consisted of the general population, (3) the variables in the original study included dietary intake of various types of vitamins, (4) the outcome of the study was the prevalence of periodontal disease, (5) multivariate-adjusted relative risk (RR) or odds ratio (ORs) along with their corresponding 95% confidence intervals (CIs) (or data allowed for the calculation of these metrics) regarding the relationship between vitamin intake and periodontal disease was provided, (6) the study conducted in either Chinese or English languages. The exclusion criteria for the article were as follows: (1) the study type of the article was not explicitly stated, (2) low-quality article that effective outcome data cannot be extracted, (3) literature with repeated data, (4) document unable to access the full text.

### Data extraction and quality assessment

Two researchers initially conducted literature screening and data extraction according to the inclusion and exclusion criteria, then cross-checked, and any differences would be resolved through discussion. The extracted contents were as follows: (1) basic information about the included studies, including the name of the first author, year of publication, type of research design, gender, age, continent and country of the research subjects, sample size, and number of cases; (2) the types and assessment methods of vitamins, the definition of periodontal disease, multivariate-adjusted ORs with 95% CIs, and confounders.

The Newcastle–Ottawa Scale (NOS) and the Agency for Healthcare Research and Quality (AHRQ) were respectively applied to assess all included case–control studies and cross-sectional studies [12, 13]. The NOS has a maximum score of 9 points and a minimum score of 0 points, ratings of “ < 4 points”, “4–6 points”, and “7–9 points” indicate “low methodological quality”, “moderate methodological quality”, and “high methodological quality” respectively. Moreover, the cross-sectional measurement standard recommended by the AHRQ consists of a total of 11 items, accumulating to 11 points, the evaluation includes three categories: “yes”, “no”, and “unclear”. A “yes” response is assigned 1 point, while a “no” or “unclear” response receives 0 points, scores of “ < 4 points”, “4–7 points”, and “8–11 points” respectively signify “low methodological quality”, “medium methodological quality”, and “high methodological quality”.

### Statistical analysis

The multivariate-adjusted ORs and their corresponding 95% CI were initially compiled based on the extracted information, and an Excel Macro file was utilized to convert the effect sizes used in different reference groups in the original studies to be consistent with the lowest group as the reference [14]. Subsequently, the Cochrane Q test and *I*^*2*^ were jointly used to test the heterogeneity among the studies, if the heterogeneity was *P* > 0.1 and *I*^*2*^ < 50%, a fixed effect model would be employed for the meta-analysis, while if the heterogeneity was *P* ≤ 0.1 or *I*^*2*^ ≥ 50%, a random effect model was used. Leave-one-out sensitivity analysis was used to explore the influence of each study on the overall results by removing each study individually [15]. Finally, the funnel plot was utilized to assess whether there was publication bias, and if publication bias was detected, the trim and fill method of the funnel plot was employed for further correction. All data were analyzed using Stata V.16.0 (Stata Corp, College Station, Texas, United States), and statistical significance was determined by a bilateral *P* < 0.05.

## Results

### Literature screening process and results

**Fig. 1:**
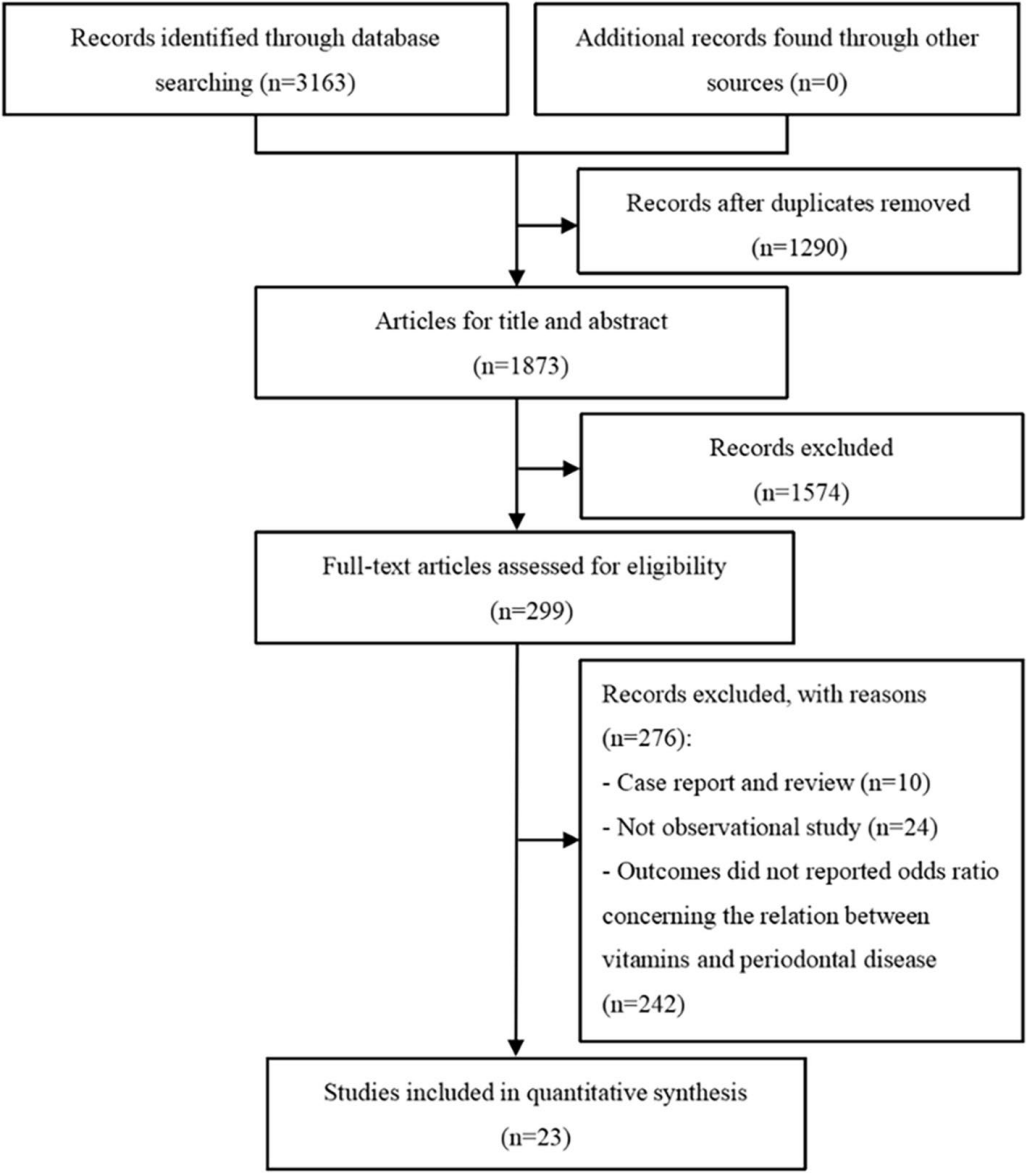
Flowchart of literature search

A preliminary search yielded a total of 3163 relevant articles. After layer-by-layer screening, 23 English documents that met the criteria were ultimately included [7–10, 16–34] (Fig. 1).

### Basic characteristics and quality assessment

Table 1 presents the basic characteristics of the included studies. The five case–control studies were all scored between 6–7 (Supplementary Table 1), and the scores of eighteen cross-sectional studies were all 7–8 (Supplementary Table 2), which indicated that all included studies were of moderate to high quality.

**Table 1:** Characteristics of the included studies in the meta-analysis

First author (Year)	Country (Continent)	Study design	Gender, Age(years)	Sample size (Cases)	Exposure assessment	Definition for periodontal disease	Types of vitamins: OR (95% CI)	Adjustment for covariates
Chapple (2007) [16]	America (North America)	Cross-sectional study	Both, > 20	11480 (609)	Blood sample	≥ 2 mesiobuccal sites with CAL ≥ 5mm and ≥ 1 mesiobuccal sites with PD ≥ 4mm	A: 0.92(0.85–1.00) C:0.76(0.69–0.84) E:0.97(0.90–1.05)	age, gender, race/ethnicity, cigarette smoking, oral contraceptives /hormone replacement therapy use, diabetes, poverty-income ratio, and education
Yu (2007) [17]	America (North America)	Cross-sectional study	Both, > 60	844 (125)	Blood sample	≥ 10% of sites with CAL ≥ 4mm and ≥ 10% sites with PD ≥ 3mm	Folate: 0.74(0.59–0.93)	age, sex, race, educational level, body mass index, bleeding on probing and probing sites
Alshouibi (2013) [18]	America (North America)	Cross-sectional study	Men, 62,9	562 (98)	FFQ	≥ 2 sites with CAL ≥ 6mm and ≥ 1 sites with PD ≥ 5 mm(not on same tooth)	D:0.97(0.96–0.98)	age, sex, race, educational level, body mass index, bleeding on probing and probing sites
Millen (2013) [32]	America (North America)	Cross-sectional study	Women, 66.6	920 (612)	Blood sample	≥ 2 interproximal sites with CAL ≥ 4mm (not on same tooth) or ≥ 2 interproximal sites with PD ≥ 5mm (not on same tooth), alveolar crest height and tooth loss	D:0.67(0.47–0.95)	age, frequency of dental visits, and body mass index
Lee (2015) [20]	Korea (Asia)	Cross-sectional study	Both, > 19	6011, 1938	Blood sample	(CPI) ≥ 1 sites with PD ≥ 3.5mm	D:0.98(0.79–1.22)	age, gender, educational level, and household income, use of floss, use of interproximal toothbrush, alcohol consumption, diabetes, obesity, and dietary conditions
Antonoglou (2015) [19]	Finland (Europe)	Case–control study	Both, -	85 (55)	Blood sample	PD ≥ 4mm and CAL ≥ 4mm	D:0.97(0.95–1.00)	age, plaque, smoking, high-density lipoprotein cholesterol, body mass index, and gender
Antonoglou (2015) [33]	Finland (Europe)	Cross-sectional study	Both, 30–49	1205 (32)	Blood sample	PD ≥ 4mm	D:0.99(0.99–1.00)	gender, age, education, presence of dental plaque, number of teeth, dental attendance pattern, tooth brushing frequency, lipid medication, body mass index and alcohol consumption
Abreu (2016) [21]	America (North America)	Case–control study	Both, 35–64	48 (24)	Blood sample	≥ 2 interproximal sites with CAL ≥ 4mm or ≥ 2 interproximal sites with PD ≥ 5mm	D:0.885(0.785–0.997)	age, gender, and body mass index
Adegboye (2016) [34]	Danish (Europe)	Cross-sectional study	Both, 18–95	3287 (316)	FFQ	≥ 2 interproximal sites with CAL ≥ 6mm and ≥ 1 interproximal sites with PD ≥ 5mm	D:0.85(0.66–1.01)	age, gender, education, smoking, sucrose intake, alcohol consumption, number of teeth, daily brushing, regular visits to the dentist, and chronic illness.
Huang (2017) [22]	America (North America)	Cross-sectional study	Both, -	754 (173)	24-hDR	BOP and ≥ 2 sites with CAL ≥ 4mm	D:0.979(0.964–0.995)	age, gender, smoking status, and physical activity
Park (2017) [24]	Korea (Asia)	Cross-sectional study	Both, 19–39	2049 (279)	24-hDR	(CPI) ≥ 1 sites with PD ≥ 3.5mm	A: 0.85(0.61–1.21) C: 0.77(0.55–1.08)	age, gender, household income, education, daily frequency of tooth brushing, use of floss /interdental brush, smoking status and pack-years, diabetes mellitus and obesity
Lee (2017) [9]	Korea (Asia)	Cross-sectional study	Both, > 19	10930	24-hDR	(CPI) PD ≥ 3.5mm	C: 0.86(0.77–0.96)	age, sex, income, tooth brushing, use of floss, dental visit, alcohol drinking, smoking, diabetes, hypercholesterolemia, hypertension and obesity
Laky (2017) [23]	Austria (Europe)	Case–control study	Both, 35.4	58 (29)	Blood sample	≥ 5 teeth with PD ≥ 5mm	D: 0.68(0.50–0.88) E: 0.53(0.28–0.99)	age, gender, and smoking
Yoon (2017) [25]	Korea (Asia)	Cross-sectional study	Both, > 65	1021 (446)	Blood sample	(CPI) PD ≥ 4mm	D: 0.58(0.34–0.98)	gender, education, income, living area, the frequency of tooth-brushing, use of oral hygiene, oral health check-ups, missing teeth, drinking, smoking experience, body mass index, regular physical activity, and walking practice
Luo (2018) [26]	America (North America)	Cross-sectional study	Both, ≥ 30	6415 (2950)	24-hDR	≥ 2 interproximal sites with CAL ≥ 4mm (not on same tooth) or ≥ 2 interproximal sites with PD ≥ 5mm (not on same tooth)	A: 0.56(0.44–0.72) B1: 0.75(0.58–0.97) B2: 0.83(0.64–1.07) B6: 0.89(0.71–1.12) Folate: 0.80(0.64–1.00) B12: 0.90(0.71–1.15) C: 0.71(0.58–0.89) D: 0.86(0.68–1.10) E: 0.63(0.49–0.82)	age, gender, ethnicity, education, income-to-poverty ratio, smoking, history of diabetes mellitus, hypertension, smoking, days per week using floss, and body mass index
Ketharanathan (2019) [7]	Norway (Europe)	Case–control study	Both, 30–70	92 (48)	Blood sample	≥ 2 sites with CAL ≥ 6mm and ≥ 1 sites with PD ≥ 5mm	D: 0.96(0.94–0.99)	age and race
Kim (2020) [27]	Korea (Asia)	Cross-sectional study	Both, ≥ 50	5405 (1289)	Blood sample	≥ 2 sites with CAL ≥ 6mm and ≥ 1 sites with PD ≥ 5mm	D: 0.66(0.42–1.05)	age, sex, month of blood collection, body mass index, smoking status, alcohol consumption status, regular exercise, antihypertensive medication, and antidiabetic medication
Alzahrani (2021) [28]	Saudi (Asia)	Case–control study	Both, 30–48	123 (60)	Blood sample	≥ 2 interproximal sites with CAL ≥ 4mm (not on same tooth) or ≥ 2 interproximal sites with PD ≥ 5mm (not on same tooth)	D: 0.964(0.931–0.999)	age, gender, and body mass index
Zhou (2021) [29]	America (North America)	Cross-sectional study	Both, 50	2928 (301)	Blood sample	≥ 2 interproximal sites with CAL ≥ 6mm (not on same tooth) and ≥ 1 interproximal site with PD ≥ 5mm (not on same tooth)	D: 0.75(0.63- 0.89)	gender, hypertension, direct HDL-Cholesterol, age, race/Hispanic origin, glycohemoglobin, education level, diabetes mellitus, body mass index, marital status, creatinine, health insurance, smoking status, and ratio of family income to poverty
Hosoda (2021) [10]	Japan (Asia)	Cross-sectional study	Both, 20.4	120 (49)	DHQ	(CPI)PD ≥ 4mm	Folate: 0.99(0.97–1.01) C: 1.03(0.99–1.07) E: 0.53(0.28–0.99)	body mass index, presence of snacks, and dietary hardness
Li (2022) [30]	America (North America)	Cross-sectional study	Both, ≥ 30	5530 (3994)	24-hDR	≥ 2 interproximal sites with CAL ≥ 4mm or ≥ 2 interproximal sites with PD ≥ 5mm	A: 0.835(0.694–1.004) B1: 1.084(0.977–1.203) B2: 0.916(0.841–0.999) C: 1.130(1.039–1.228) E: 0.968(0.953–0.984)	age, gender, educational level, income, body mass index, diabetes, hyperlipidemia and hypertension
Li (2022) [31]	America (North America)	Cross-sectional study	Both, ≥ 30	5145 (-)	24-hDR	≥ 2 interproximal sites with CAL ≥ 3mm (not on the same tooth), and ≥ 2 interproximal sites with PD ≥ 4mm or one site with PD ≥ 5mm (not on same tooth)	C: 0.93(0.78–1.12)	age, sex, race, had at least 12 alcohol drinks/one year, body mass index, hypertension, hyperlipidemia, education level, marital status, annual household income, diabetes history, general health condition, number of flossing / weeks, sleep disorder, smoked at least 100 cigarettes in life and energy
Watson (2022) [8]	America (North America)	Cross-sectional study	Both, 56.2	9476 (1634)	24-hDR	painful gums and/or bleeding gums and/or loose teeth	B6: 0.83(0.71–0.96) Folate: 0.81(0.69–0.94) B12: 0.83(0.71–0.97) C: 0.77(0.66–0.90) D: 0.89(0.77–1.04) E: 0.63(0.69–0.94)	age, sex, ethnicity, Townsend Deprivation Index, body mass index, smoking status, and alcohol drinking status

*FFQ* food frequency questionnaire, *DHQ* self-administered diet history questionnaire, *24-hDR* 24-h dietary record, *PD* probing depth, *CAL* clinical attachment loss, *CPI* community periodontal index

### The relationship between vitamin intake and the risk of periodontal disease

#### Vitamin A

**Fig. 2:**
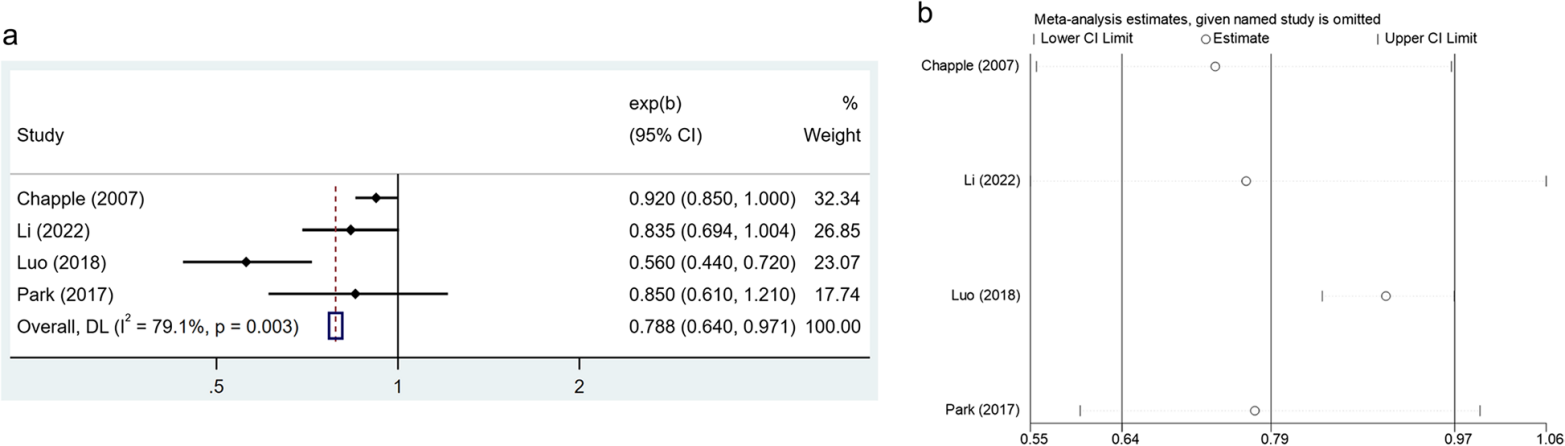
**a** Forest plot of the relationship between vitamin A consumption and the prevalence of periodontal disease. **b** Sensitivity analysis of the association between vitamin B complex intake and periodontal disease

Four studies reported the vitamin A intake in 17,642 healthy individuals and 7832 periodontal disease patients. Due to the statistically significant heterogeneity between studies (*I*^*2*^ = 79.1%, *p* = 0.003), a random effect model was performed. The results indicated that there was a negative correlation between higher levels of vitamin A intake and the risk of periodontal disease (OR: 0.788, 95% CI: 0.640–0.971) (Fig. 2a). Leave-one-out sensitivity analysis was performed to explore the impact of a single study on the comprehensive results, indicating that the overall results did not change substantially after excluding any one study (Fig. 2b).

#### Vitamin B complex

**Fig. 3:**
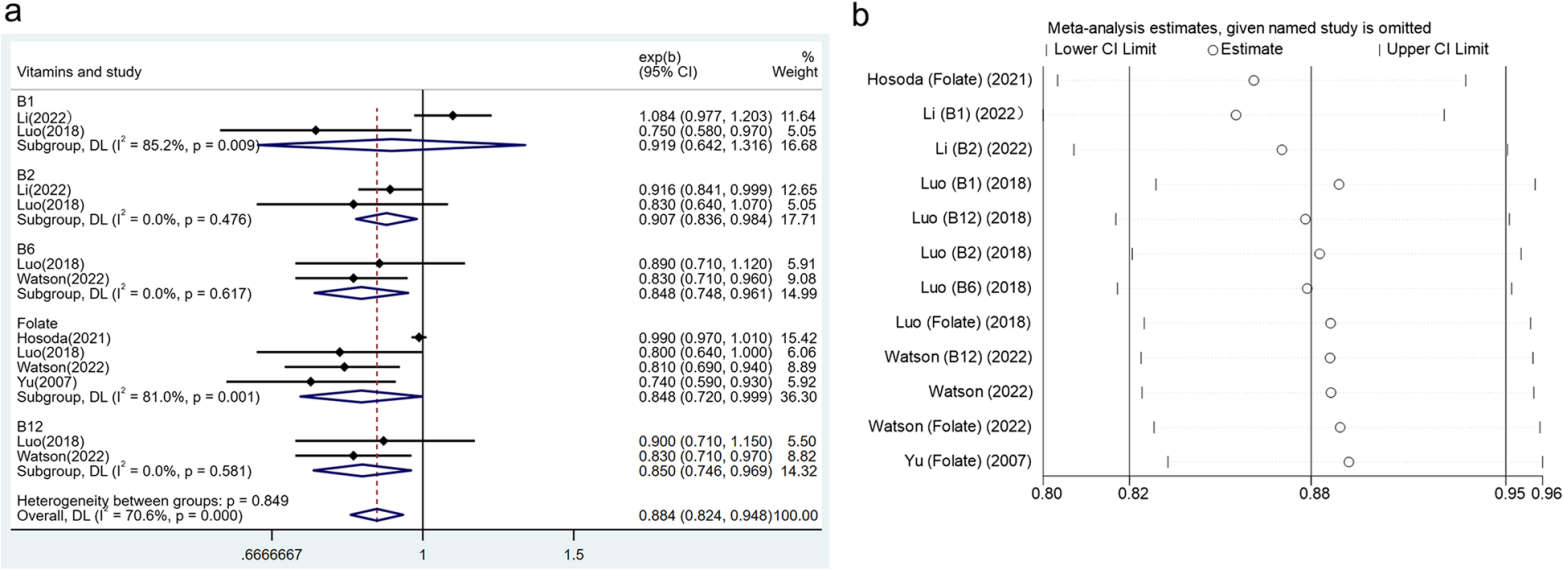
**a** Forest plot of the relationship between vitamin B complex consumption and the prevalence of periodontal disease. **b** Sensitivity analysis of the association between vitamin B complex intake and periodontal disease

A total of twelve effect groups from five articles provided information on vitamin B complex intake. The overall results revealed that a higher level of vitamin B complex was related to the decrease in the prevalence of periodontal disease (OR: 0.884, 95% CI: 0.824–0.948) (Fig. 3a). Meanwhile, high-dose vitamin B2 (OR: 0.907, 95% CI: 0.836–0.984), vitamin B6 (OR: 0.848, 95% CI: 0.748–0.961), vitamin B12 (OR: 0.850, 95% CI: 0.746–0.969), and folate (OR: 0.848, 95% CI: 0.720–0.999) consumption alone also displayed a negative correlation with periodontal disease, but no significant association was found in the subgroup of solely intaking high-dose vitamin B1 (OR: 0.919, 95% CI: 0.642–1.316). No statistically significant heterogeneity among studies was observed (*I*^*2*^ = 70.6%, *p* < 0.001). In the sensitivity analysis, after excluding each study separately, no substantial alteration was observed in the overall research results (Fig. 3b).

#### Vitamin C

**Fig. 4:**
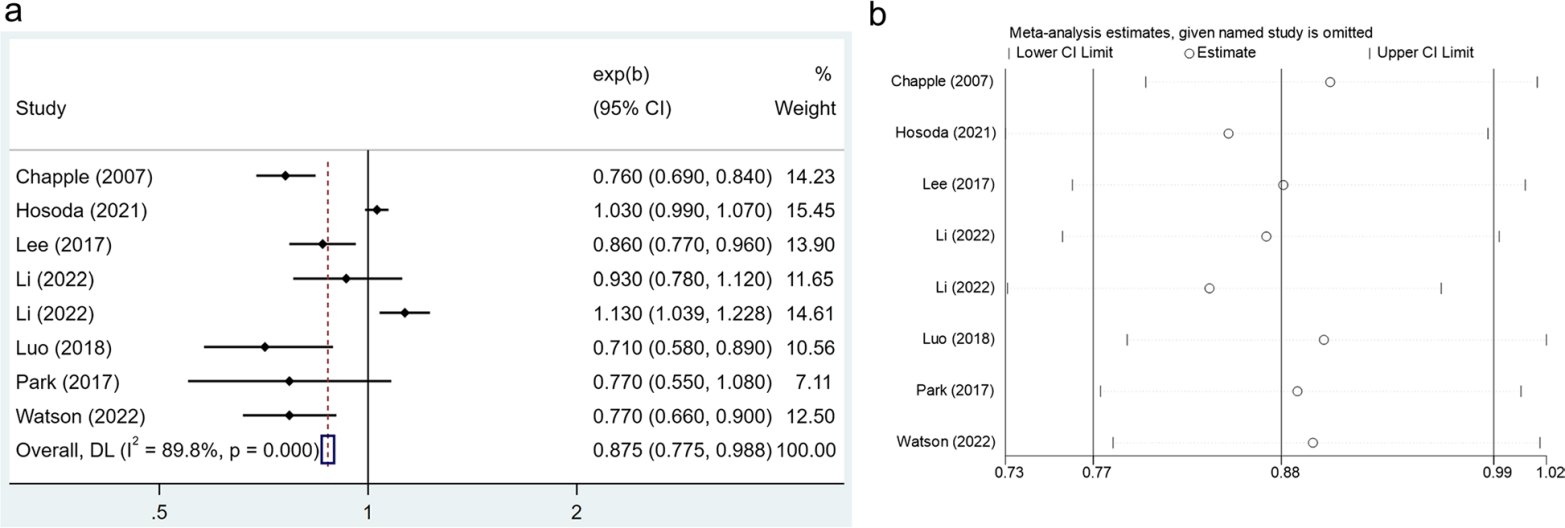
**a** Forest plot of the relationship between vitamin C consumption and the prevalence of periodontal disease. **b** Sensitivity analysis of the association between vitamin C intake and periodontal disease

As regards vitamin C dietary intake, eight studies with 51,145 participants suggested that a higher level of vitamin C consumption was negatively correlated with periodontal disease (OR: 0.875, 95% CI: 0.775–0.988) (Fig. 4a), with statistically significant between-study heterogeneity (*I*^*2*^ = 89.8%,* p* < 0.001). Moreover, the overall research findings did not undergo substantial changes when each study was excluded separately in the sensitivity analysis (Fig. 4b).

#### Vitamin D

**Fig. 5:**
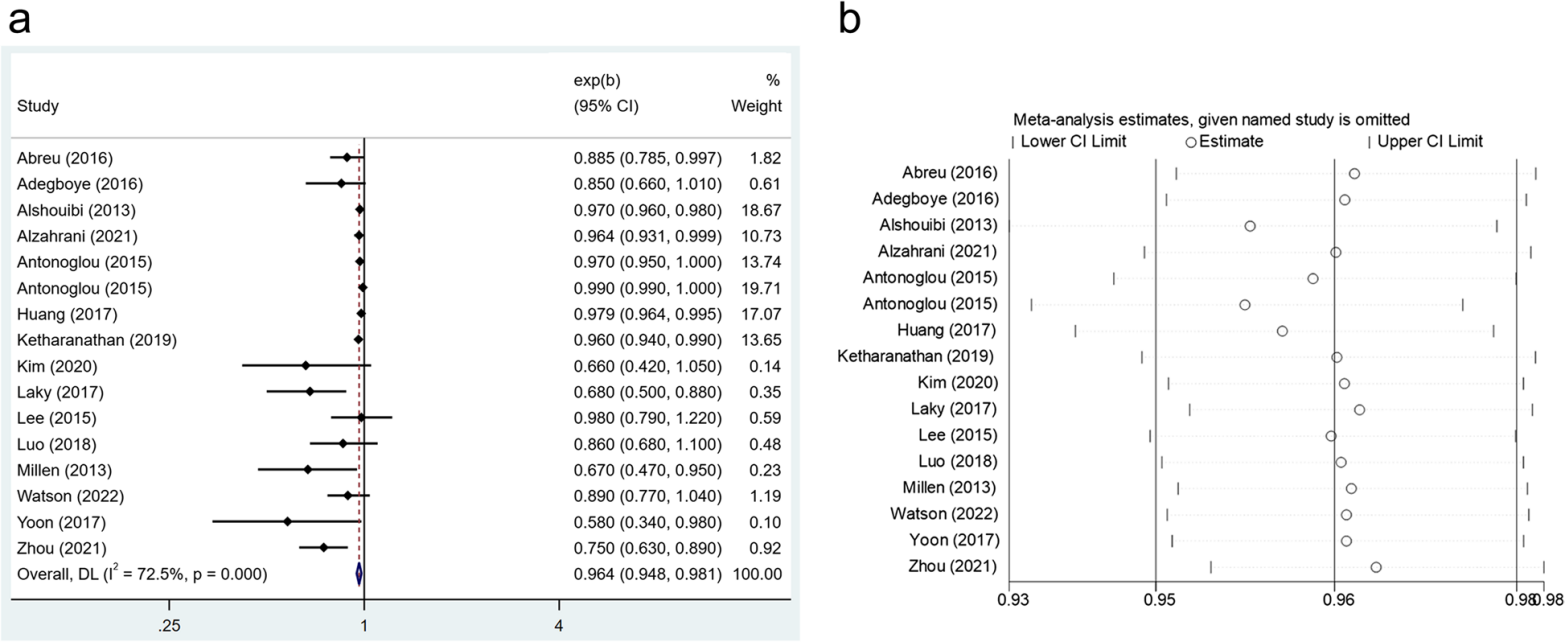
**a** Forest plot of the relationship between vitamin D consumption and the prevalence of periodontal disease. **b** Sensitivity analysis of the association between vitamin D intake and periodontal disease

The meta‐analysis of sixteen studies revealed that a higher dose of vitamin D was associated with a lower rate of periodontal disease (OR: 0.964, 95% CI: 0.948–0.981) (Fig. 5a). No statistically significant heterogeneity was found among studies (*I*^*2*^ = 72.5%, *P* < 0.001). Furthermore, leave-one-out sensitivity analysis demonstrated the total results remained unchanged, after excluding each study separately (Fig. 5b).

#### Vitamin E

**Fig. 6:**
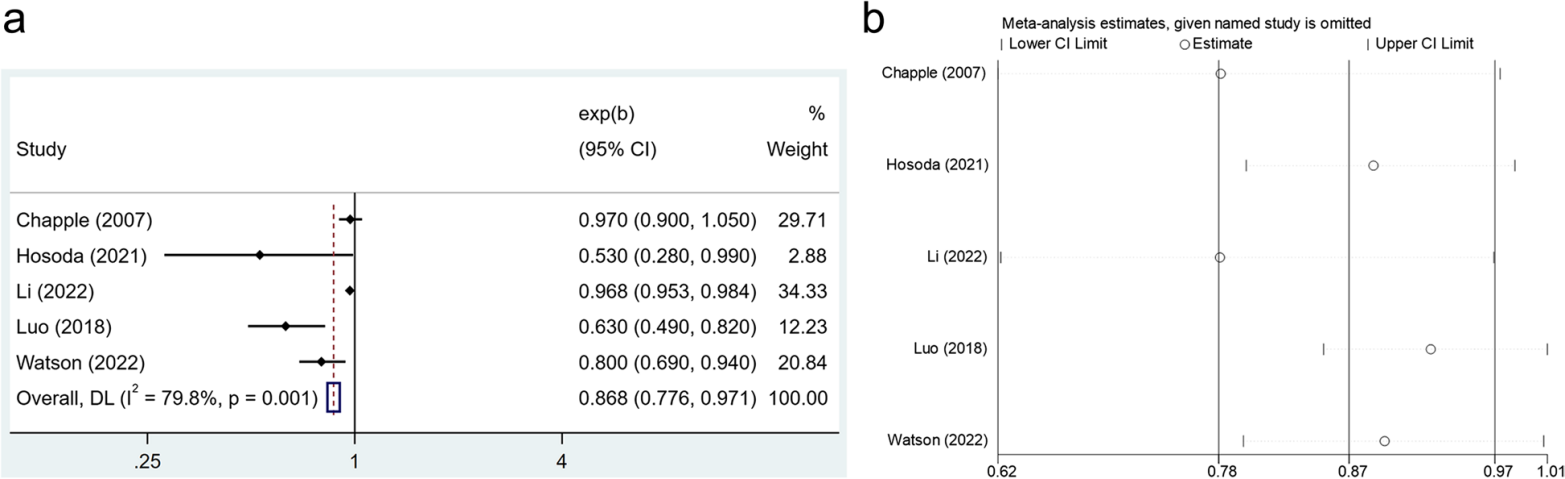
**a** Forest plot of the relationship between vitamin E consumption and the prevalence of periodontal disease. **b** Sensitivity analysis of the association between vitamin E intake and periodontal disease

Five studies offered data on vitamin E, and the results, based on a random‐effect model, indicated that there was an inverse association between the higher vitamin E intake dose and periodontal disease (OR: 0.868,95% CI: 0.776–0.971) (Fig. 6a), with statistically significant heterogeneity (*I*^*2*^ =79.8%, *P* < 0.001). Besides, we found that,after removing any one of the studies, the collective results did not change significantly in the sensitivity analysis (Fig. 6b).

### Publication bias

**Fig. 7:**
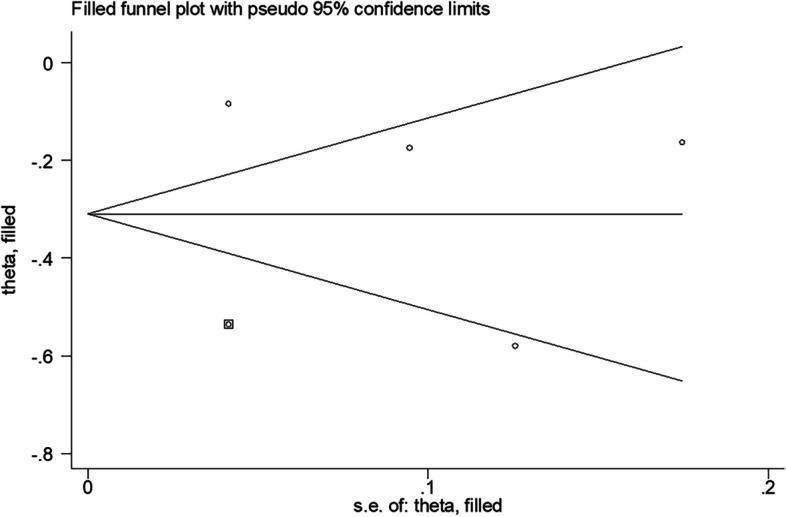
Filled funnel plot with pseudo 95% confidence limits about the analysis of vitamin A and periodontal disease

The funnel plots were used to assess publication bias for all polled effect sizes. Five results all indicated significant asymmetry. Thus, we further performed the trim-and-fill method of the funnel plot to correct for any potential missing studies, but no missing articles were detected except for the overall results of vitamin A. One article was filled in the analysis of vitamin A and periodontal disease, which led to publication bias in this meta-analysis, and the correlation between the two became more significant after adjustment (OR: 0.734, 95% CI: 0.571–0.946) (Fig. 7). The overall results of the other four meta-analyses remained unchanged, indicating that there was no obvious publication bias in the analysis of vitamin B complex, vitamin C, vitamin D, and vitamin E consumption with the prevalence of periodontal disease (Supplementary file 1).

## Discussion

A total of 45 effect sizes from 23 observational studies, with a total of 74,488 study participants, were included to explore the relationship between various types of vitamin consumption and the risk of periodontal disease. The results based on the random effect model showed that higher levels of vitamin A, vitamin B complex, vitamin C, vitamin D, and vitamin E intake were associated with the reduced prevalence of periodontal disease. In sensitivity analysis, the total findings of five meta-analyses all remained substantively unchanged, indicating that this study's results were robust and reliable.

Lots of previous studies have also investigated the relationship between various types of vitamin intake and the rate of periodontal disease [35]. Luo et al. also found that there was a significant negative correlation between adequate intake of vitamin A, vitamin B1, folate, vitamin C, and vitamin E and the risk of periodontal disease [26]. Machado et al.'s meta-analysis also indicated that, compared with the periodontal health group, the levels of vitamin D were significantly lower in the periodontitis group [36]. Our finding results were consistent with these studies. Nevertheless, Park et al. point out that the levels of vitamin A, and vitamin C were significantly lower in the periodontitis group than in the periodontal health group [24]. Li et al. proposed that the vitamin C intake was 158.49 mg, the incidence of periodontitis seemed to be the lowest, and inadequate or excessive intake of vitamin C could boost the likelihood of developing periodontitis [30].

The mechanism underlying the association between periodontal disease and vitamins is still unclear, but current research mainly focuses on the anti-inflammatory and reparative effects of vitamins on periodontal tissue. In terms of anti-inflammatory effects, vitamins can inhibit the development of bacterial infections in periodontal tissue by enhancing the phagocytic function and immune chemotaxis of neutrophils and macrophages. For example, vitamin D increases the phagocytic function and immune chemotaxis of macrophages and enhances the activity of 1-α-hydroxylase in monocytes, thereby enhancing the immune response of periodontal tissue [37]. Additionally, vitamins also exert anti-inflammatory effects by inhibiting the production of excessive reactive oxygen species (ROS) and reducing the synthesis of inflammatory mediators and bacterial toxins. Vitamin D reduces the production of cytokines, such as IL-17, by helper T cells (Th), which is associated with an increase in the incidence of periodontitis [38]. Moreover, high levels of vitamin D can also reduce the levels of inflammatory molecules, such as RANKL, IL-1, or IL-6 [39, 40]. Vitamin E not only exerts antioxidant effects by reducing lipid peroxidation and increasing the level of superoxide dismutase [41, 42] but also reduces the expression of IL-6 and IL-1β in gingival fibroblasts, thereby reducing the production of ROS [43]. Additionally, vitamin E also inhibits the activity of lipopolysaccharides from Porphyromonas gingivalis and stimulates the growth and migration of gingival fibroblasts [44]. Vitamin C reduces the apoptotic and cytotoxic effects of Porphyromonas gingivalis on human gingival fibroblasts [45].

In terms of periodontal tissue repair and regeneration, vitamins have the functions of increasing collagen synthesis and promoting bone metabolism, thereby improving periodontal tissue regeneration and alveolar bone mineralization levels. Vitamin A has the ability to stimulate the proliferation, migration, and osteogenic differentiation of human periodontal ligament cells, thus being considered as having the potential to promote periodontal tissue regeneration [46]. Supplementation of vitamin B complex may improve wound healing ability and help increase clinical attachment levels in patients with chronic periodontitis undergoing periodontal surgery [47]. Vitamin C may increase the quantity of collagen fibers and the strength of blood vessels, thereby promoting periodontal tissue regeneration [48]. Oral supplementation of vitamin C improves postoperative healing in patients with chronic periodontitis and those undergoing guided bone regeneration or bio-oss collagen transplantation for implant surgery [49]. Vitamin D improves the absorption of calcium and phosphate by the intestines and kidneys, directly enhances the mineralization ability of alveolar bone, and inhibits the synthesis and release of parathyroid hormone, thereby suppressing the activity of osteoclasts and reducing alveolar bone resorption [50].

This meta-analysis had several notable strengths. First, to the best of our knowledge, it was the first meta-analysis to investigate the relationship between multiple vitamins and the risk of periodontal disease. Previous studies had mainly focused on exploring the association between a single type of vitamin and periodontal disease. It is important to consider that, in our daily diet, the intake of different types of vitamins is not isolated, and there may be interactions and synergistic effects between different vitamins. Therefore, we believed that conducting a combined multivitamin analysis provided a more comprehensive approach to exploring the correlation between vitamins and periodontal disease, thus, seeking the underlying etiology of periodontal disease. Second, it should be noted that the inclusion of a large number of participants and multiple countries in this meta-analysis has enabled us to obtain more robust and stable results. Third, all the studies included have adjusted different confounding factors, which increased the credibility of this study. Considering the high prevalence of periodontal disease in the population, this study might have important implications for the development of primary preventive measures and serve as evidence to support the formulation of dietary guidelines for individuals.

The main limitation of the present study was all the articles included were observational studies, so the causal relationship between vitamin intake levels and periodontal disease could not yet be explained. Another disadvantage is that due to the inconsistent methods used to measure vitamins and the varying highest or lowest intake levels for vitamins among these epidemiological studies, we were unable to suggest a suitable range for vitamin consumption.

## Conclusions

The results of this meta-analysis indicated a negative correlation between the higher level of vitamin A, B complex, C, D, and E intake and the risk of periodontal disease. Therefore, this provides new avenues for clinical prevention, where healthcare professionals can enhance awareness about appropriate vitamin supplementation in oral health education to prevent and reduce the occurrence of periodontal disease. Besides, future research should focus on more prospective studies and randomized controlled trials to explore the causal relationship between the two and further investigate the role of vitamin intervention in the treatment of periodontal disease. Additionally, we encouraged future research to adopt uniform diagnostic criteria for periodontal disease and standardized methods for measuring vitamin levels to conduct higher-quality studies, which will provide more assistance in the development of clinical and public health-related measures.

### Supplementary Information

**Additional file 1: Supplementary Table 1.** The quality assessment scale of case-control study.

**Additional file 2: Supplementary Table 2.** The quality assessment scale of cross-sectional study.

**Additional file 3.**

## Data Availability

All data generated or analysed during this study are included in this published article [and its supplementary information].

## Abbreviations

ORs: Odds ratio
CIs: Confidence intervals
FFQ: Food frequency questionnaire
DHQ: Self-administered diet history questionnaire
24-hDR: 24-Hour dietary record
PD: Probing depth
CAL: Clinical attachment loss
CPI: Community periodontal index
ROS: Reactive oxygen species
