# Supplementary material for: Vitamin intake and periodontal disease: a meta-analysis of observational studies
Source: BMC Oral Health. 2024 Jan 20;24:117. doi: 10.1186/s12903-024-03850-5 (PMC10799494; doi:10.1186/s12903-024-03850-5)
Supplement: Supplementary file 3 — Additional file 3. [file 12903_2024_3850_MOESM3_ESM.docx]

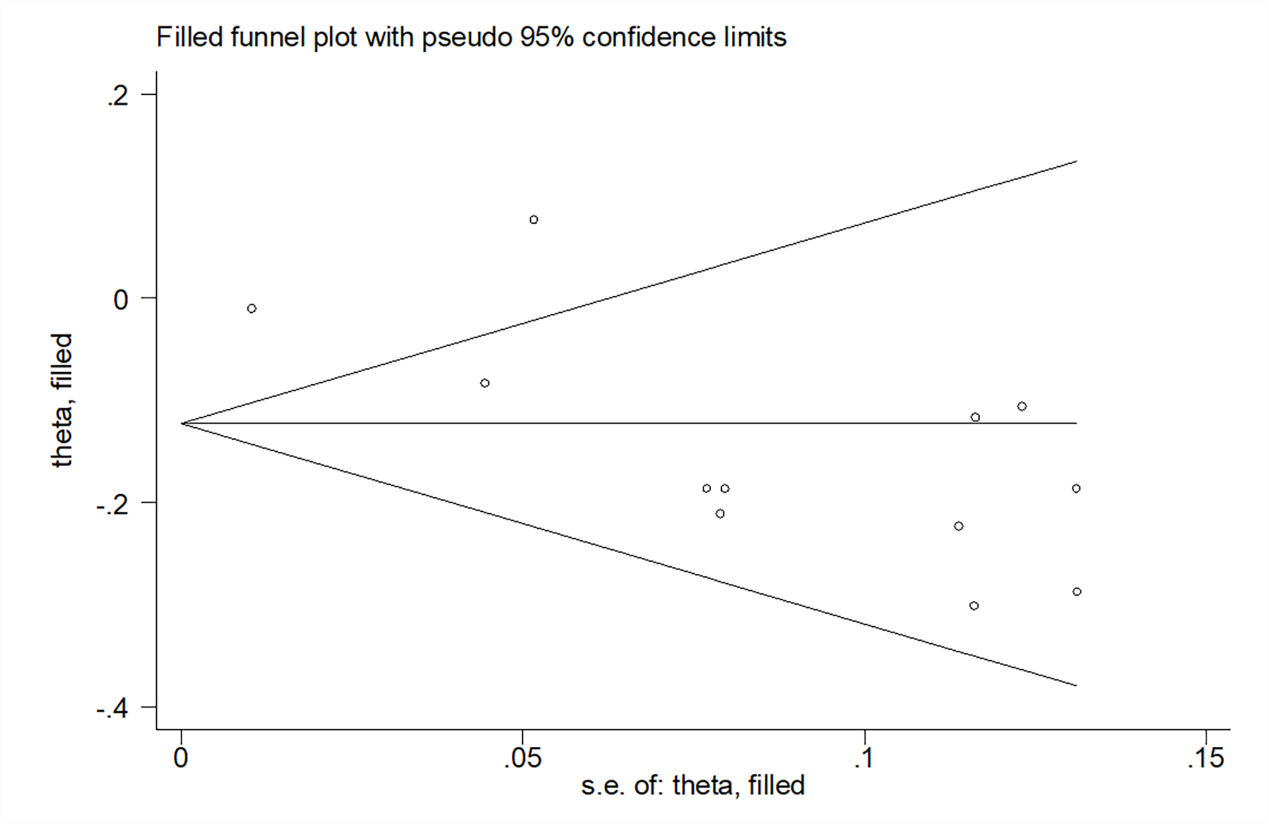


(1)Filled funnel plot with pseudo 95% confidence limits about the analysis of vitamin B complex and periodontal disease.


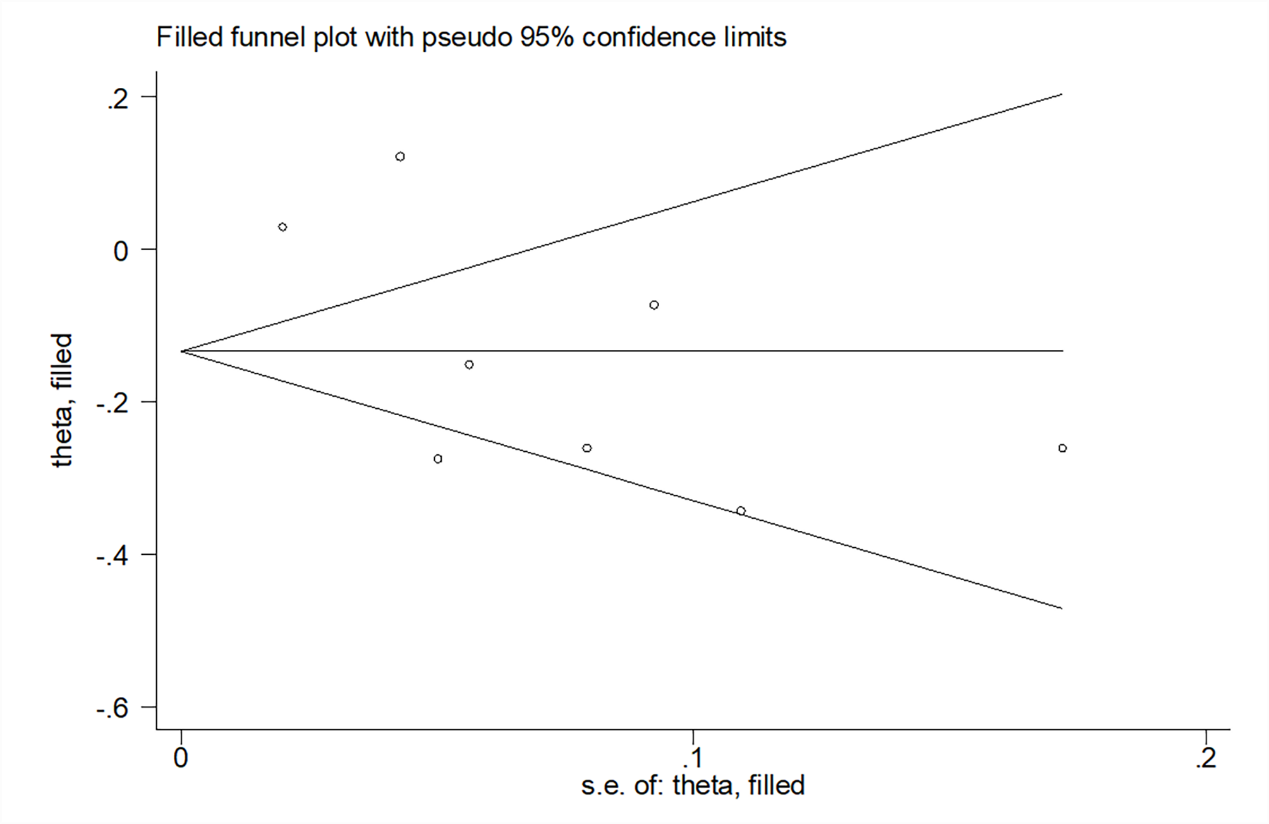


1. Filled funnel plot with pseudo 95% confidence limits about the analysis of vitamin C and periodontal disease.


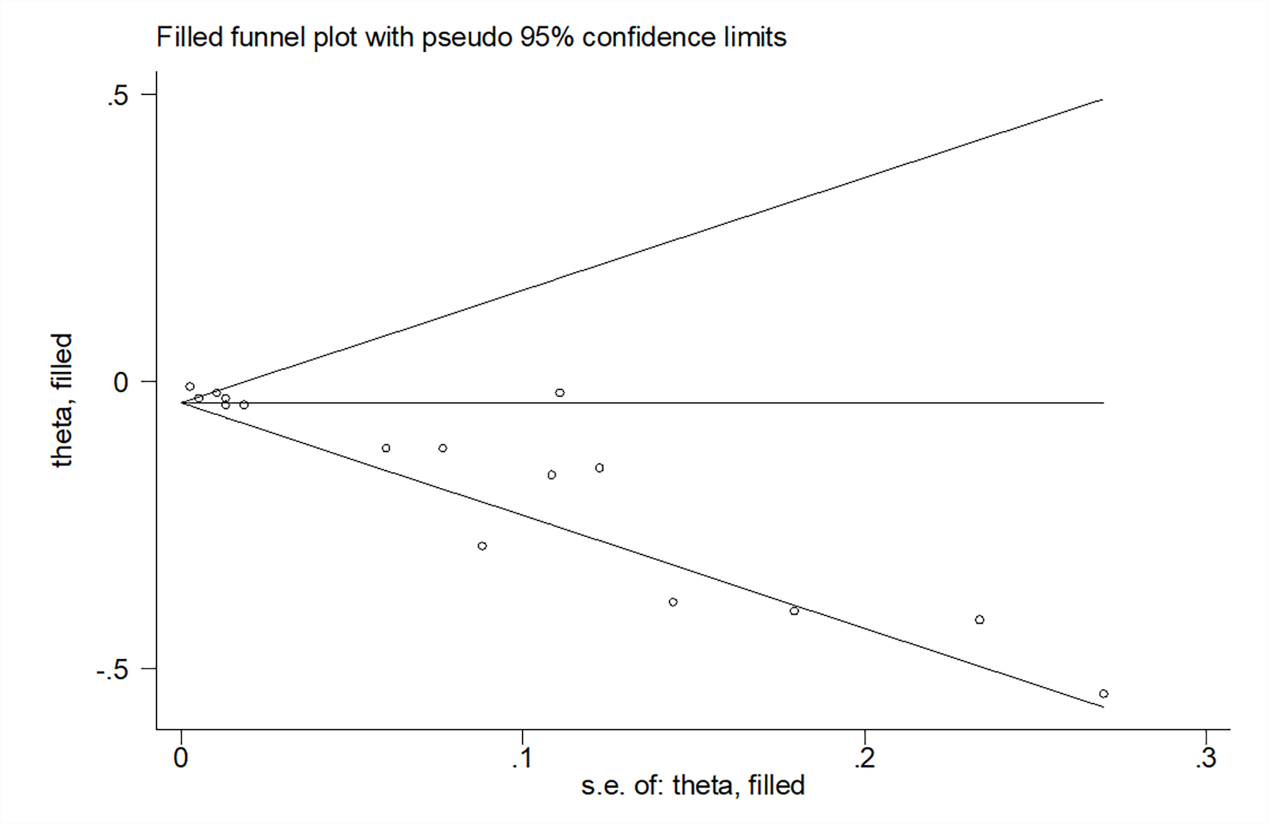


1. Filled funnel plot with pseudo 95% confidence limits about the analysis of vitamin D and periodontal disease.


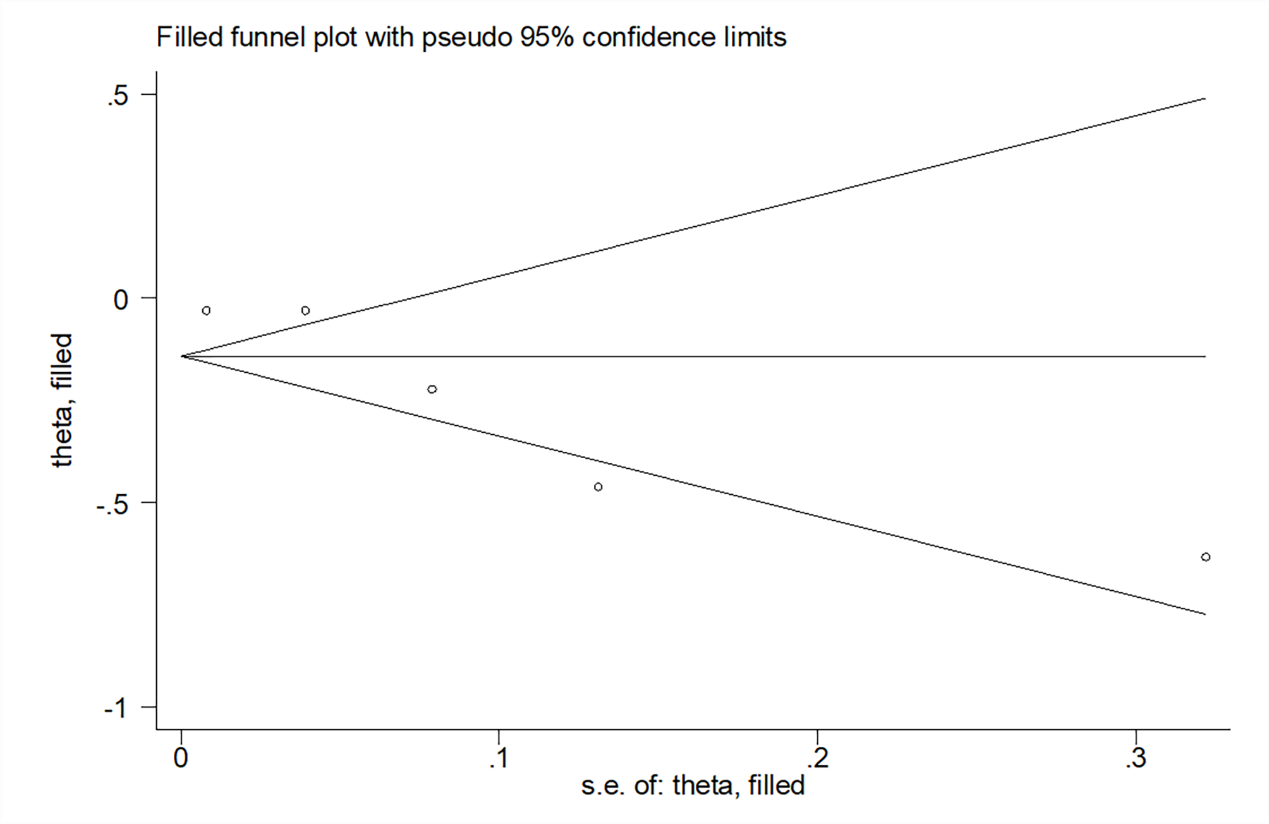


(4) Filled funnel plot with pseudo 95% confidence limits about the analysis of vitamin E and periodontal disease.
